# Acute and Subacute Oral Toxicity of Mumefural, Bioactive Compound Derived from Processed Fruit of *Prunus mume* Sieb. et Zucc., in ICR Mice

**DOI:** 10.3390/nu12051328

**Published:** 2020-05-07

**Authors:** Jungim Kim, Mira Han, Won Kyung Jeon

**Affiliations:** 1 Herbal Medicine Research Division, Korea Institute of Oriental Medicine, 1672 Yuseong-daero, Daejeon 34054, Korea; imlee1001@kiom.re.kr (J.K.); mira0221@kist.re.kr (M.H.); 2 Convergence Research Center for Diagnosis, Treatment and Care System of Dementia, Korea Institute of Science and Technology, 5 Hwarang-ro 14-gil, Seongbuk-gu, Seoul 02792, Korea

**Keywords:** mumefural, single oral dose toxicity, repeated oral dose toxicity, ICR mice

## Abstract

Mumefural is a bioactive compound derived from the processed fruit of *Prunus mume* Sieb. et Zucc., a traditional health food; however, its safety has not been evaluated. We investigated the toxicity of mumefural through single and repeated oral administration at doses of 1250, 2500, and 5000 mg/kg in Institute of Cancer Research (ICR) mice. The acute toxicity assessment was not associated with adverse effects or death. Similarly, the subacute (four weeks) toxicity assessment did not reveal any mumefural-associated mortality, abnormal organ damage, or altered clinical signs, body weight, food consumption, or hematological parameters. However, albumin/globulin ratio and chloride ion levels were significantly increased in male mice treated with mumefural at ≥2500 mg/kg. Female mice exhibited significantly higher levels of chloride, sodium, and potassium ions, at a dose of 5000 mg/kg. Furthermore, the administration of 2500 and 5000 mg/kg mumefural decreased the absolute weight of spleen in male mice. These findings indicated that the approximate lethal dose of mumefural in ICR mice was >5000 mg/kg. No significant mumefural toxicity was observed at ≤5000 mg/kg. Our findings provide a basis for conducting future detailed studies to evaluate reproductive, neurological, genetic, and chronic toxicity of mumefural.

## 1. Introduction

The fruit of *Prunus mume* Sieb. et Zucc. is valuable in East Asia, where it has been consumed as a healthy food and traditional medicine for thousands of years [1,2]. It is used in side dishes, teas, drinks, and traditional medicines that improve digestion and bowel movement and exhibit anti-pyretic action [3]. Recently, the use of herbs and botanicals as food for specified health uses (FOSHU) or as nutraceuticals has increased globally, and the value of the fruit of *P. mume* has increased [4]. 

In recent decades, *P. mume* fruit extracts (PMFE) have been reported to have various beneficial effects, such as the improvement of blood circulation [5] and energy metabolism [6]. It also has anticancer [7,8], antioxidant [9,10], anti-inflammatory [11,12], and anti-microbial [13] activity. We reported that Fructus mume, the processed fruit of *P. mume*, effectively improves cognitive impairment in familial Alzheimer’s disease [14], scopolamine-induced memory impairment [15], and chronic cerebral hypoperfusion-induced cognitive impairment [11,12]. 

Mumefural is a bioactive citric acid derivative found in the heated fruit of *P. mume* [16]. It is listed in the Human Metabolome Database (HMDB) as a nutrient and classified as a tricarboxylic acid derivative [17]. Mumefural has been identified as a phosphodiesterase 4D (PDE4D) inhibitor [18], as well as a gamma-amino butyric acid (GABA) and benzodiazepine (BZ) agonist [19]. It also has beneficial effects, such as the improvement of blood fluidity [16] and the inhibition of pandemic influenza A virus [20]. Furthermore, we reported that mumefural improves cognitive impairment induced by chronic cerebral hypoperfusion via improved cholinergic system and the neuroprotective effect [21].

Consequently, mumefural has attracted attention as a dietary supplement, leading to the development of methods for its isolation, quantitative analysis, and synthesis [20,22,23]. However, the safety of mumefural has still not been assessed. Therefore, we investigated the toxicity of mumefural after single or repeated oral administration for four weeks in Institute of Cancer Research (ICR) mice, to determine its safety profile.

## 2. Materials and Methods 

### 2.1. Test Material

Mumefural (CAS No. 222973-44-6) was purchased from U-Chem (Gyeonggi-do, Republic of Korea). The dose formulations were prepared on the day of administration. Mumefural was weighed and suspended in distilled water by vortexing and sonication.

### 2.2. Experimental Animals

All experimental animal protocols were approved by the Institutional Animal Care and Use Committee of Nonclinical Research Institute, ChemOn Inc. (Serial No.: 18-M754 and 18-M846). Female and male CrljOri:CD1(ICR) mice were purchased from Orient Bio Inc. (Gyeonggi-do, Republic of Korea). Male mice were housed individually and no more than three female mice were housed in a stainless-steel cage (W 165 × L 240 × H 145 mm) and provided wood gnawing blocks as an enrichment product for improved welfare. The room environment was maintained under standard controlled conditions: temperature, 23 ± 3 °C; relative humidity, 55 ± 15%; ventilation, 10–20 air changes/h; and 150–300 Lux of luminous intensity on a 12-h light/dark cycle. 

### 2.3. Acute Toxicity Study

Healthy animals were randomly divided in groups of three males or three females each. After a 3–4 h starvation, animals were administered 1250, 2500, or 5000 mg/kg mumefural solution (prepared by dissolution in sterile distilled water) or the vehicle (sterile distilled water). Food was provided for approximately 1–2 h after administration. All mice were continuously monitored for changes in urination, defecation, vocalization, breathing, surprise reaction, ptosis, gait disturbance, edema, sensory abnormalities, aggression, muscle tone, trauma, and hair loss or mortality 1 h after administration of mumefural and every 6 h after that. The day of dosing was designated as day 0 and animals were observed until day 14. Each mouse was weighed on day 0, 1, 3, 7, and 14. On day 14, all survivors were anesthetized by inhalation of carbon dioxide (CO_2_) and euthanized by exsanguination via the posterior vena cava and abdominal aorta. The skin, brain, and all internal organs in the abdominal and thoracic cavities were excised and observed macroscopically.

### 2.4. Subacute (4 Weeks) Toxicity Study

The subacute toxicity study was selected based on the results of the acute toxicity study. ICR mice with body weights close to the mean value were selected and randomized into groups of five males and five females each, which were administered 1250, 2500, and 5000 mg/kg mumefural, or the vehicle once a day for 4 weeks.

#### 2.4.1. General Observations

During the experimental period, all animals were monitored daily for mortality and changes in clinical signs. The body weight and food consumption were recorded on day 1, and once a week after that. Pre-weighed food was provided in each cage and the remainder was weighed the next day to calculate the mean daily consumption (g/day). 

#### 2.4.2. Clinical Pathology

All animals were starved for approximately 4 h without restricting water consumption prior to necropsy and blood sample collection. Blood samples were drawn from the posterior vena cava under deep isoflurane (Kyongbo Pharm. Co., Ltd., Seoul, Republic of Korea) anesthesia. The animals were given 2%–3% isoflurane (depending on the condition of the animal) in a mixture of oxygen and nitrogen (3:7) in an anesthesia chamber and monitored while blood was being collected. For tge hematological analysis, approximately 0.3 mL of blood was placed in a CBC bottle (Becton Dickinson, NJ, USA) containing anti-coagulant tripotassium ethylenediaminetetraacetic acid (K3-EDTA). Then, the white blood cell (WBC) count, red blood cell (RBC) count, hemoglobin (HGB) concentration, hematocrit (HCT), mean corpuscular volume (MCV), mean corpuscular hemoglobin (MCH), mean corpuscular HGB concentration (MCHC), RBC distribution width (RDW), HGB distribution width (HDW), platelet (PLT) volume, mean PLT volume (MPV), neutrophil (NEU), lymphocyte (LYM), monocyte (MONO), eosinophil (EOS), basophil (BASO), and large unstained cells (LUC) were detected using a coulter counter (ADVIA 2120, Siemens, NY, USA). 

For the clinical biochemistry analysis, 0.5 mL of blood was collected into a 5 mL Vacutainer tube (Becton Dickinson, Oxford, UK). To obtain the serum, blood samples were centrifuged at 3000 rpm for 10 min. The following parameters were measured by using an AU680 serum biochemistry analyzer (Beckman Coulter, Tokyo, Japan): aspartate aminotransferase (AST), alanine aminotransferase (ALT), alkaline phosphatase (ALP), creatine phosphokinase (CPK), total bilirubin (TBIL), glucose (GLU), total cholesterol (TCHO), triglyceride (TG), total protein (TP), albumin (ALB), albumin/globulin ratio (A/G), blood urea nitrogen (BUN), creatinine (CRE), inorganic phosphorus (IP), calcium ion (Ca^2+^), potassium ion (K^+^), sodium ion (Na^+^), and chloride ion (Cl^−^).

#### 2.4.3. Necropsy and Organ Weight Measurements

At the end of the 4-week subacute toxicity study, blood samples were collected under anesthesia with 2%–3% isoflurane, then animals were euthanized and gross necropsy was performed. The skin, brain, and all internal organs in the abdominal and thoracic cavities were excised and observed macroscopically. The absolute organ weights were measured using an electronic balance (SECURA224-1S, Sartorius AG, Germany), and relative organ weights (organ weight/body weight) were calculated for the adrenal gland, thymus, spleen, kidney, heart, lung, brain, and liver with gall bladder.

### 2.5. Statistical Analysis

The data were statistically analyzed using the statistical package for the social sciences (SPSS) version 22.0 software (IBM, Armonk, NY, USA). The data of body weights, food consumption in males, hematological parameters, clinical biochemistry test, and organ weights were assumed to be normally distributed and analyzed using a one-way analysis of variance (ANOVA). The assumption of homogeneity of variance was tested using the Levene’s test. If the overall ANOVA was significant and the assumption of homogeneity of variance was met, Duncan’s multiple range test was used as a post hoc test, to determine the groups that were significantly different from the negative control group. If the group sample sizes were not equal, Scheffe test was used instead. If the assumption of homogeneity of variance was not met, Dunnett T3 test was used as a post hoc test. Food consumption in female mice from the two groups was compared using Student’s *t*-test. The data are presented as means ± standard deviation (SD) and a *p* < 0.05 was considered statistically significant. 

## 3. Results

### 3.1. Acute Toxicity Study

**Figure 1:**
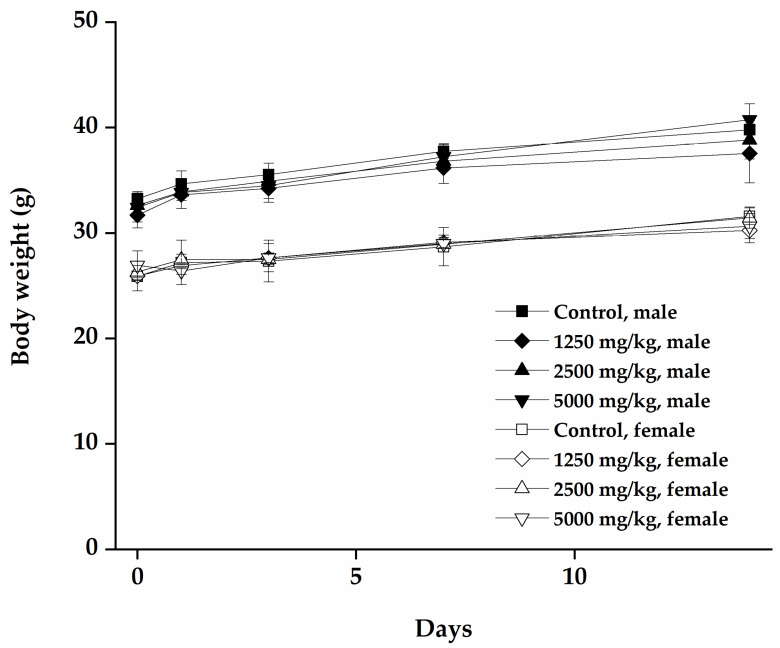
Body weight change in mice during 15 days, following single oral administration of mumefural. Change in body weight was measured for 14 days after single administration of mumefural. Data are expressed as means ± SD.

In the preliminary study, to determine the solubility of mumefural in sterile distilled water, the high-dose of mumefural set at 5000 mg/kg and the two lower doses at 2500 and 1250 mg/kg were selected to test the safety of mumefural. During the 14-day observation period, all mice were healthy with no mortality, mumefural-related clinical signs (Table 1), or changes in body weight (Figure 1, Table 2). In addition, macroscopic examination did not reveal any notable necropsy finding such as bleeding, or change in color or size of any organs. On day 1, piloerection and locomotor activity decreased in one female mouse. Moreover, on day 1 and 2, squeak and respiration rate increased in the same mouse (Table 1). These clinical signs were transient, and the mouse ultimately recovered after day 3. Based on these observations, the approximate lethal dose (ALD) of mumefural after single dose oral administration was >5000 mg/kg in both sexes of ICR mice, and it is considered a non-toxic chemical based on the Globally Harmonized System of Classification and Labelling of Chemicals (GHS) classification [24]. 

### 3.2. Subacute (4 Weeks) Toxicity Study

#### 3.2.1. Mortality and Clinical Signs

In the repeated oral dose toxicity study, no mumefural-related mortalities were observed at all three dose levels tested, except for one female mouse in the 2500 mg/kg group. On day 2 of the experiment, one female mouse died in the 2500 mg/kg treated group (Table 3), although it exhibited no symptoms before death. Macroscopic examination of the heart, lung, and liver at necropsy showed a dark brown discoloration. Oral gavage is associated with increased stress-induced mortality rate of 15% [25,26]. The death of this female mouse can thus be attributed to errors in oral administration caused by increased stress due to inadequate skills. Abnormal clinical signs were also observed in one male mouse in the 1250 mg/kg treated group, which constantly squeaked from day 12 to day 14 (Table 3). This abnormality was temporary and involved only one mouse; therefore, it was not considered to be related to mumefural. The data for this mouse was excluded from the toxicity study analysis. There were no meaningful changes in clinical signs (Table 3).

#### 3.2.2. Changes in Body Weight and Food Consumption

**Figure 2:**
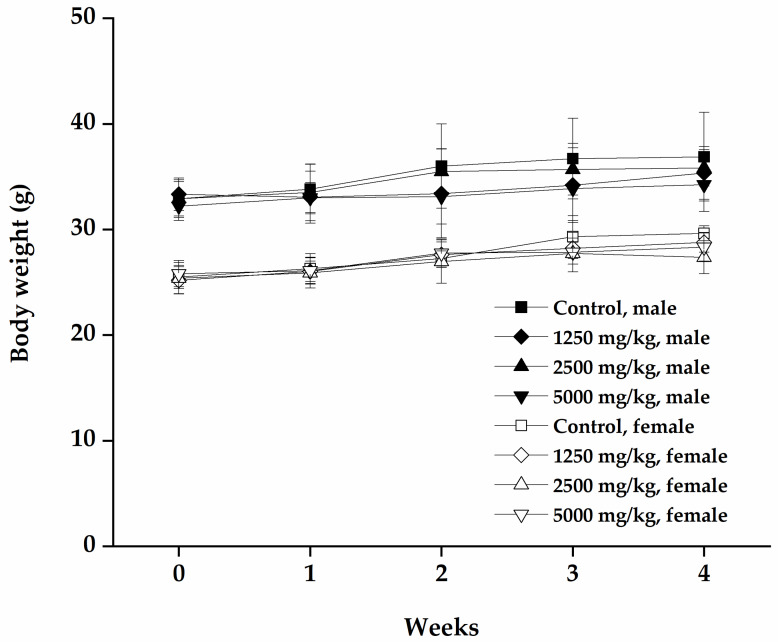
Body weight change in mice during repeated oral dose administration of mumefural for 4 weeks. Change in body weight was measured for 4 weeks after single administration of mumefural. Data are expressed as means ± SD.

The body weight of each mouse increased in a time-dependent manner (Figure 2). The body weight gain was highest in the vehicle control group compared with the mumefural-treated groups, and female mice administered 5000 mg/kg showed significantly lower body weights (Table 4, *p* < 0.05), without meaningful changes in food consumption, compared with the vehicle control group (Table 5). The mean daily food consumption of female mice administered 1250 mg/kg was significantly lower on day 27 compared with the vehicle control group (Table 5, *p* < 0.01). This change was not dose-dependent and occurred at different doses of mumefural in the female group only. Therefore, it cannot be concluded that the body weight gain and the change of food consumption were correlated.

#### 3.2.3. Hematology and Serum Biochemistry

Mice of both sexes administered mumefural showed normal ranges of hematology parameters (Table 6). In the mumefural-administered male mice, the A/G and Cl^−^ levels in the clinical biochemistry results were increased compared with those of the vehicle control group (Table 7, *p* < 0.05, *p* < 0.01). The Cl^−^ level was significantly higher in female mice administered 2500 and 5000 mg/kg mumefural than in the vehicle control mice (*p* < 0.05). The Na^+^ level was significantly higher in female mice treated with 5000 mg/kg mumefural, compared with the vehicle control group (*p* < 0.05). Furthermore, the TP level of males administered 2500 mg/kg and the K^+^ level of female mice administered 5000 mg/kg were significantly lower compared with the vehicle control group (both *p* < 0.05) (Figures S1 and S2).

#### 3.2.4. Relative Organ Weights and Necropsy

The absolute spleen weights of male mice were dose-dependently lower than those of the vehicle group (Table 8, *p* < 0.05). However, female mice were not affected. No mumefural-related changes were observed in the necropsy, and one male mouse in the vehicle control group showed a decrease in thymus size (Table 9, Figure S3).

## 4. Discussion

Mumefural is receiving attention as a bioactive compound that supports and promotes good health and, thus, its toxicity should be considered to ensure safe oral intake. Previous toxicity studies have been reported for products containing mumefural, but the toxicity of mumefural has not been investigated. Previous studies have reported the safe oral dose of fructus mume extract to be >5000 mg/kg, following single oral administration to ICR mice [27]. In addition, a clinical study conducted in a Japanese population revealed that PMFE containing 1% mumefural showed anti-hypertensive effects without abnormal changes in physical, hematological, and biological parameters when 3 g/day PMFE was consumed [28]. In this study, we investigated the toxicity of mumefural in ICR mice, for the first time. Our results provide a rational basis to select the dose levels for a subsequent repeated oral dose toxicity study.

Single oral administrations of 1250, 2500, and 5000 mg/kg did not cause any observable adverse effects or mortality in both male and female mice. Only one female mouse in the 5000 mg/kg treated group showed transient abnormalities. A daily oral administration of up to 5000 mg/kg mumefural for 4 weeks did not cause critical toxicities. Nevertheless, minor and transient changes were observed in some mumefural-administered mice.

In the subacute study, the body weight gain was statistically lower in female mice in the group treated with the highest dose of mumefural than it was in female mice in the control group. However, food consumption, hematological liver (AST, ALT, and ALP) and kidney (BUN and CRE) function indices, and other toxicity parameters, were within normal ranges in our study. 

The results of this experiment indicate that the hematopoietic system was normal in all the mice. Serum biochemistry parameters including A/G ratio, Cl^−^, Na^+^, K^+^, and TP levels were altered after mumefural administration. However, the changes did not exceed the normal range. The kidney regulates the volume of various body fluid compartments, fluid osmolality, acid-base balance, various electrolyte concentrations, and the removal of toxins [29]. Increased level of Cl^−^ showed dose-dependency in mice of both sexes. Moreover, the changes in Na^+^ and K^+^ concentrations were significant in the highest dose of mumefural-administered female mice. 

The spleen is the largest organ and plays a role in immune cell balance [30]. Decreased absolute spleen weight was observed in male mice treated with mumefural in a dose-dependent manner. However, the hematological parameters (such as RBC, WBC, and monocyte counts), which are involved in the immune system were normal and spleen percentage to body weight did not differ. Moreover, mumefural did not affect the organ weight of female mice. 

Our study demonstrated changes in clinical and biochemical parameters in experimental animals following single and 4-week administration of mumefural, but further chronic toxicity studies of mumefural following long-term administration would be necessary. In addition, histological changes in the organs induced by administration of mumefural will need to be investigated and further chronic toxicity studies are recommended to ensure the safety of mumefural.

## 5. Conclusions

In this study, to the best of our knowledge, we conducted the first toxicity tests of mumefural, a bioactive compound found in various functional foods and supplements. Our findings indicate that the ALD of mumefural in ICR mice was >5000 mg/kg, the highest dosage tested, which is recommended for further toxicity studies to determine any toxicologically relevant effects. Finally, the use of mumefural to support a healthy life style require it to possess a good level of safety, and this study will provide a foundation for future comprehensive studies to build on. 

## Figures and Tables

**Table 1:** Clinical signs in acute toxicity study of mumefural in mice.

	Control	1250 mg/kg	2500 mg/kg	5000 mg/kg
Male				
No observable abnormality	3/3	3/3	3/3	3/3
Female				
No observable abnormality	3/3	3/3	3/3	2/3
Increased respiration rate	0/3	0/3	0/3	1/3
Squeak	0/3	0/3	0/3	1/3
Piloerection	0/3	0/3	0/3	1/3
Decreased locomotor activity	0/3	0/3	0/3	1/3

Number of mice showing change in clinical sign/total number of mice.

**Table 2:** Body weight gain in acute toxicity study of mumefural in mice.

	Dose (mg/kg)	Survival ^a^	Mean Body Weight (g)			Final Weight Relative to Control (%)
			Initial	Final	Change	
Male						
	Control	3/3	33.2 ± 0.69	39.8 ± 1.11	6.6 ± 0.42	
	1250	3/3	31.7 ± 1.22	37.5 ± 2.79	5.8 ± 2.20	94.22
	2500	3/3	32.6 ± 0.80	38.8 ± 1.81	6.2 ± 2.61	97.49
	5000	3/3	32.4 ± 1.38	40.8 ± 1.49	8.3 ± 1.37	102.51
Female						
	Control	3/3	25.9 ± 1.40	31.6 ± 0.79	5.7 ± 0.69	
	1250	3/3	25.9 ± 0.42	30.2 ± 1.18	4.3 ± 1.13	95.57
	2500	3/3	26.3 ± 0.58	31.4 ± 1.00	5.1 ± 1.24	99.97
	5000	3/3	26.92 ± 1.38	30.6 ± 1.18	3.7 ± 0.21	96.84

Data are expressed as means ± SD. ^a^ Number of mice showing change in clinical sign/total number of mice.

**Table 3:** Clinical signs in subacute toxicity study of mumefural in mice.

	Control	1250 mg/kg	2500 mg/kg	5000 mg/kg
Male				
No observable abnormality	5/5	4/5	5/5	5/5
Constantly squeaked	0/5	1/5	0/5	0/5
Female				
No observable abnormality	5/5	5/5	4/5	5/5
Death	0/5	0/5	1/5	0/5

Number of mice showing change in clinical sign/total number of mice.

**Table 4:** Body weight gain in subacute toxicity study of mumefural in mice.

	Dose (mg/kg)	Survival ^a^	Mean Body Weight (g)			Final Weight Relative to Control (%)
			Initial	Final	Change	
Male						
	Control	5/5	32.9 ± 1.63	36.9 ± 4.20	4.0 ± 2.89	
	1250	5/5	33.3 ± 1.54	35.4 ± 2.49	2.0 ± 1.03	95.93
	2500	5/5	32.9 ± 1.76	35.8 ± 1.73	2.9 ± 2.46	97.02
	5000	5/5	32.2 ± 1.36	34.2 ± 2.53	2.0 ± 3.60	92.68
Female						
	Control	5/5	25.5 ± 1.11	29.6 ± 0.70	4.1 ± 0.71	
	1250	5/5	25.2 ± 1.29	28.8 ± 0.93	3.6 ± 0.46	90.18
	2500	4/5	25.4 ± 1.48	27.4 ± 1.57	1.5 ± 1.80	92.57
	5000	5/5	25.8 ± 1.25	28.3 ± 1.34	2.5 ± 0.68 *	95.61

Data are expressed as means ± SD. * Significant difference at *p* < 0.05 compared with vehicle control. ^a^ Number of mice showing change in clinical sign/total number of mice.

**Table 5:** Food consumption in subacute toxicity study of mumefural in mice.

Day	Control	Food Consumption (g/day)		
		1250 mg/kg	2500 mg/kg	5000 mg/kg
Male				
1	5.4 ± 0.91	5.4 ± 0.77	5.3 ± 0.66	4.6 ± 2.12
7	4.7 ± 1.00	5.4 ± 0.85	5.1 ± 0.33	5.2 ± 0.65
14	4.4 ± 0.31	4.8 ± 1.23	5.2 ± 1.23	4.3 ± 0.73
21	5.0 ± 0.82	5.2 ± 0.23	4.2 ± 1.07	4.6 ± 0.23
27	4.6 ± 0.62	5.5 ± 1.00	4.5 ± 0.76	4.6 ± 0.63
Female				
1	5.1 ± 0.57	3.4 ± 0.65	3.8 ± 2.97	3.7 ± 0.53
7	4.5 ± 0.04	4.9 ± 0.25	4.0 ± 0.34	5.0 ± 0.79
14	4.4 ± 0.54	4.4 ± 0.63	3.8 ± 0.05	4.5 ± 0.68
21	4.5 ± 0.80	3.2 ± 0.21	4.1 ± 0.65	4.5 ± 0.73
27	4.6 ± 0.06	3.8 ± 0.07 *	3.7 ± 0.24	5.0 ± 1.07

Data are expressed as mean ± SD. * Significant difference at *p* < 0.05 compared with vehicle control group.

**Table 6:** Hematology data of subacute toxicity study of mumefural in mice.

	Control	1250 mg/kg	2500 mg/kg	5000 mg/kg
Male				
WBC (10^3^/μL)	1.26 ± 0.92	1.37 ± 0.26	1.26 ± 0.56	0.96 ± 0.43
RBC (10^6^/μL)	8.70 ± 0.75	9.00 ± 0.49	9.17 ± 0.34	8.88 ± 0.28
HGB (g/dL)	13.9 ± 1.0	14.4 ± 0.7	14.2 ± 0.8	14.3 ± 0.3
HCT (%)	44.8 ± 3.6	45.8 ± 2.0	45.8 ± 1.7	45.8 ± 0.8
MCV (fL)	51.5 ± 0.6	50.9 ± 1.4	50.0 ± 0.9	51.6 ± 0.8
MCH (pg)	16.0 ± 0.4	16.0 ± 0.6	15.4 ± 0.3	16.1 ± 0.4
MCHC (g/dL)	31.1 ± 0.5	31.4 ± 0.5	30.9 ± 0.6	31.1 ± 0.5
RDW (%)	12.1 ± 0.3	12.3 ± 0.6	12.1 ± 0.8	12.3 ± 0.4
HDW (g/dL)	2.05 ± 0.09	2.11 ± 0.09	2.06 ± 0.07	2.12 ± 0.13
PLT (10^3^/μL)	1130.6 ± 165.3	1231.4 ± 144.5	1236.0 ± 153.9	1190.0 ± 148.2
MPV (fL)	4.88 ± 0.33	4.86 ± 0.40	4.82 ± 0.31	4.88 ± 0.28
NEU (%)	18.8 ± 14.7	18.4 ± 6.8	23.3 ± 6.4	21.4 ± 6.2
LYM (%)	66.1 ± 25.8	73.6 ± 12.4	67.9 ± 7.0	73.3 ± 6.2
MONO (%)	0.60 ± 0.29	3.24 ± 5.69	1.42 ± 1.07	0.70 ± 0.24
EOS (%)	13.88 ± 23.73	4.30 ± 2.96	6.30 ± 6.38	3.80 ± 1.56
BASO (%)	0.12 ± 0.08	0.06 ± 0.05	0.12 ± 0.08	0.02 ± 0.04
LUC (%)	0.40 ± 0.16	0.38 ± 0.13	0.90 ± 0.80	0.76 ± 1.20
Female				
WBC (10^3^/μL)	2.11 ± 1.23	2.00 ± 0.64	2.00 ± 1.17	2.59 ± 2.19
RBC (10^6^/μL)	9.00 ± 0.39	9.20 ± 0.69	8.72 ± 0.23	9.04 ± 0.53
HGB (g/dL)	14.5 ± 0.8	14.4 ± 0.7	13.9 ± 0.2	14.3 ± 0.9
HCT (%)	45.9 ± 1.9	46.1 ± 2.7	45.2 ± 0.8	45.6 ± 2.9
MCV (fL)	51.1 ± 0.6	50.2 ± 1.2	51.8 ± 1.0	50.4 ± 1.4
MCH (pg)	16.1 ± 0.5	15.7 ± 0.5	15.9 ± 0.4	15.8 ± 0.6
MCHC (g/dL)	31.6 ± 0.8	31.4 ± 0.5	30.8 ± 0.7	31.5 ± 0.4
RDW (%)	12.6 ± 0.5	12.4 ± 0.4	13.0 ± 0.2	13.3 ± 0.8
HDW (g/dL)	2.13 ± 0.07	2.14 ± 0.08	2.20 ± 0.08	2.17 ± 0.06
PLT (10^3^/μL)	1292.8 ± 135.5	1165.6 ± 91.9	1084.8 ± 77.2	1218.2 ± 132.6
MPV (fL)	5.02 ± 0.55	4.90 ± 0.72	5.40 ± 0.79	5.32 ± 0.40
NEU (%)	14.3 ± 3.8	16.1 ± 3.1	16.3 ± 4.7	18.0 ± 7.4
LYM (%)	77.0 ± 6.7	78.9 ± 3.7	77.6 ± 5.0	76.4 ± 8.0
MONO (%)	0.90 ± 0.29	0.60 ± 0.25	0.95 ± 0.50	0.62 ± 0.27
EOS (%)	7.24 ± 4.39	3.96 ± 1.39	4.55 ± 0.45	4.40 ± 1.35
BASO (%)	0.24 ± 0.32	0.10 ± 0.07	0.13 ± 0.10	0.12 ± 0.08
LUC (%)	0.34 ± 0.11	0.46 ± 0.15	0.48 ± 0.10	0.48 ± 0.29

Data are expressed as means ± SD. Abbreviations: white blood cell (WBC), red blood cell (RBC), hemoglobin (HGB), hematocrit (HCT), mean corpuscular volume (MCV), mean corpuscular hemoglobin (MCH), mean corpuscular HGB concentration (MCHC), RBC distribution width (RDW), HGB distribution width (HDW), platelet (PLT), mean PLT volume (MPV), neutrophil (NEU), lymphocyte (LYM), monocyte (MONO), eosinophil (EOS), basophil (BASO), large unstained cells (LUC).

**Table 7:** Serum biological parameters in subacute toxicity study of mumefural in mice.

	Control	1250 mg/kg	2500 mg/kg	5000 mg/kg
Male				
AST (U/L)	62.6 ± 3.5	66.6 ± 26.5	60.5 ± 6.0	66.8 ± 8.5
ALT (U/L)	34.6 ± 3.4	31.4 ± 4.4	30.5 ± 7.0	37.2 ± 10.9
ALP (U/L)	71.9 ± 20.7	68.2 ± 15.5	73.3 ± 6.2	71.6 ± 20.9
CPK (U/L)	89.6 ± 36.0	104.4 ± 67.3	94.2 ± 18.9	77.2 ± 26.2
TBIL (mg/dL)	0.182 ± 0.048	0.151 ± 0.033	0.149 ± 0.036	0.145 ± 0.042
GLU (mg/dL)	205.9 ± 39.2	207.2 ± 38.0	193.2 ± 43.5	180.3 ± 62.2
TCHO (mg/dL)	217.8 ± 22.0	196.8 ± 25.7	197.2 ± 49.6	183.2 ± 50.2
TG (mg/dL)	72.0 ± 18.6	85.4 ± 31.4	58.0 ± 11.3	63.8 ± 19.2
TP (g/dL)	5.55 ± 0.14	5.33 ± 0.27	5.24 ± 0.11 *	5.28 ± 0.11
ALB (g/dL)	2.68 ± 0.11	2.69 ± 0.19	2.68 ± 0.07	2.75 ± 0.05
A/G ratio	0.94 ± 0.05	1.02 ± 0.05 *	1.05 ± 0.05 **	1.09 ± 0.07 **
BUN (mg/dL)	20.5 ± 1.4	23.3 ± 7.6	21.5 ± 6.8	19.2 ± 6.8
CRE (mg/dL)	0.28 ± 0.01	0.27 ± 0.01	0.26 ± 0.02	0.26 ± 0.03
IP (mg/dL)	8.67 ± 0.77	7.50 ± 1.19	6.59 ± 1.40	7.30 ± 1.10
Ca^2+^ (mg/dL)	9.07 ± 0.33	9.11 ± 0.22	8.92 ± 0.22	8.77 ± 0.23
Na^+^ (mmol/L)	147.7 ± 6.3	152.8 ± 0.7	149.8 ± 6.8	149.4 ± 5.5
K^+^ (mmol/L)	5.23 ± 0.35	5.45 ± 0.35	5.49 ± 0.50	4.98 ± 0.47
Cl^−^ (mmol/L)	112.9 ± 3.5	117.9 ± 1.6^*^	119.0 ± 3.9 **	117.9 ± 2.8 *
Female				
AST (U/L)	115.8 ± 83.6	85.4 ± 14.8	114.1 ± 42.3	87.4 ± 28.5
ALT (U/L)	37.0 ± 17.8	35.4 ± 13.1	49.0 ± 18.2	33.0 ± 5.1
ALP (U/L)	76.5 ± 11.8	103.9 ± 24.9	84.3 ± 3.0	89.3 ± 24.5
CPK (U/L)	94.6 ± 39.9	81.3 ± 34.9	151.3 ± 112.2	120.2 ± 50.5
TBIL (mg/dL)	0.097 ± 0.046	0.066 ± 0.036	0.093	0.083 ± 0.018
GLU (mg/dL)	190.7 ± 24.6	171.6 ± 37.6	205.4 ± 75.1	165.0 ± 28.2
TCHO (mg/dL)	137.4 ± 18.9	127.3 ± 32.2	132.0 ± 30.0	92.4 ± 22.6
TG (mg/dL)	51.2 ± 10.0	68.3 ± 16.1	60.0 ± 11.2	50.6 ± 8.1
TP (g/dL)	5.31 ± 0.03	5.50 ± 0.16	5.25 ± 0.15	5.19 ± 0.20
ALB (g/dL)	2.94 ± 0.10	3.08 ± 0.08	2.93 ± 0.11	2.91 ± 0.14
A/G ratio	1.24 ± 0.09	1.27 ± 0.05	1.26 ± 0.03	1.28 ± 0.09
BUN (mg/dL)	18.4 ± 3.7	18.8 ± 5.0	17.7 ± 5.0	19.8 ± 5.7
CRE (mg/dL)	0.28 ± 0.02	0.24 ± 0.03	0.25 ± 0.02	0.26 ± 0.01
IP (mg/dL)	8.81 ± 1.55	7.47 ± 1.37	8.59 ± 2.48	7.44 ± 0.78
Ca^2+^ (mg/dL)	9.06 ± 0.36	9.26 ± 0.39	8.94 ± 0.25	9.20 ± 0.14
Na^+^ (mmol/L)	147.3 ± 5.6	151.7 ± 2.1	152.4 ± 2.0	154.4 ± 1.4 *
K^+^ (mmol/L)	6.35 ± 0.48	5.79 ± 0.44	6.54 ± 0.94	5.14 ± 0.43 *
Cl^−^ (mmol/L)	112.6 ± 2.3	115.6 ± 1.9	120.0 ± 3.9 *	119.8 ± 2.8 *

Data are expressed as mean ± SD. * Significant difference at *p* < 0.05. ** Significant difference at *p* < 0.01 compared with vehicle control group. Abbreviations: aspartate aminotransferase (AST), alanine aminotransferase (ALT), alkaline phosphatase (ALP), creatine phosphokinase (CPK), total bilirubin (TBIL), glucose (GLU), total cholesterol (TCHO), triglyceride (TG), total protein (TP), albumin (ALB), albumin/globulin ratio (A/G), blood urea nitrogen (BUN), creatinine (CRE), inorganic phosphorus (IP), calcium ion (Ca^2+^), potassium ion (K^+^), sodium ion (Na^+^), chloride ion (Cl^−^).

**Table 8:** Absolute organ weight in subacute toxicity study of mumefural in mice.

	Control	1250 mg/kg	2500 mg/kg	5000 mg/kg
Male				
Final body weight (g)	33.97 ± 4.41	32.89 ± 2.50	33.03 ± 1.92	30.93 ± 2.58
Adrenal gland-left (% of body weight)	0.0025 ± 0.0005	0.0029 ± 0.0004	0.0027 ± 0.0002	0.0026 ± 0.0004
Adrenal gland-right (% of body weight)	0.0026 ± 0.0004	0.0026 ± 0.0004	0.0024 ± 0.0005	0.0029 ± 0.0007
Thymus (% of body weight)	0.0490 ± 0.0195	0.0462 ± 0.0108	0.0459 ± 0.0179	0.0461 ± 0.0128
Spleen (% of body weight)	0.1054 ± 0.0191	0.0900 ± 0.0169	0.0812 ± 0.0105 *	0.0796 ± 0.0079 *
Kidney-left (% of body weight)	0.2505 ± 0.0350	0.2424 ± 0.0178	0.2504 ± 0.0173	0.2309 ± 0.0206
Kidney-right (% of body weight)	0.2528 ± 0.0344	0.2528 ± 0.0211	0.2658 ± 0.0142	0.2300 ± 0.0135
Heart (% of body weight)	0.1735 ± 0.0133	0.1564 ± 0.0167	0.1593 ± 0.0038	0.1569 ± 0.0169
Lung (% of body weight)	0.2170 ± 0.0239	0.1988 ± 0.0250	0.1947 ± 0.0105	0.2061 ± 0.0194
Brain (% of body weight)	0.4821 ± 0.0204	0.4839 ± 0.0238	0.4614 ± 0.0217	0.4904 ± 0.0335
Liver (Gall bladder) (% of body weight)	1.5452 ± 0.1490	1.7238 ± 0.3261	1.5987 ± 0.1777	1.4266 ± 0.2516
Female				
Final body weight (g)	27.30 ± 1.29	26.52 ± 1.03	25.74 ± 1.19	26.18 ± 1.84
Adrenal gland-left (% of body weight)	0.0061 ± 0.0014	0.0056 ± 0.0006	0.0062 ± 0.0012	0.0057 ± 0.0010
Adrenal gland-right (% of body weight)	0.0059 ± 0.0013	0.0049 ± 0.0005	0.0052 ± 0.0010	0.0051 ± 0.0010
Thymus (% of body weight)	0.0634 ± 0.0123	0.0682 ± 0.0226	0.0531 ± 0.0142	0.0537 ± 0.0164
Spleen (% of body weight)	0.1018 ± 0.0199	0.1014 ± 0.0281	0.0899 ± 0.0105	0.0982 ± 0.0213
Kidney-left (% of body weight)	0.1589 ± 0.0164	0.1616 ± 0.0123	0.1568 ± 0.0095	0.1639 ± 0.0223
Kidney-right (% of body weight)	0.1714 ± 0.0131	0.1778 ± 0.0118	0.1616 ± 0.0089	0.1657 ± 0.0170
Heart (% of body weight)	0.1301 ± 0.0049	0.1301 ± 0.0084	0.1271 ± 0.0135	0.1286 ± 0.0159
Lung (% of body weight)	0.1890 ± 0.0192	0.1833 ± 0.0186	0.1857 ± 0.0072	0.1947 ± 0.0101
Brain (% of body weight)	0.4791 ± 0.0172	0.4730 ± 0.0302	0.4583 ± 0.0184	0.4694 ± 0.0210
Liver (Gall bladder) (% of body weight)	1.1607 ± 0.0976	1.1848 ± 0.1420	1.1380 ± 0.0999	1.1288 ± 0.0933

Data are expressed as mean ± SD. * Significant difference at *p* < 0.05 compared with vehicle control group.

**Table 9:** Summary incidence of necropsy findings in subacute toxicity study of mumefural in mice.

	Control	1250 mg/kg	2500 mg/kg	5000 mg/kg
Male				
No observable abnormality	4/5	5/5	5/5	5/5
Thymus size	1/5 (Decreased size)	0/5	0/5	0/5
Female				
No observable abnormality	5/5	5/5	4/5	5/5
Thymus size	0/5	0/5	0/4	0/5

Number of mice showing change in clinical sign/total number of mice.

## Supplementary Materials

The following are available online at https://www.mdpi.com/2072-6643/12/5/1328/s1, Figure S1. Hematological parameters (A) WBC, (B) RBC, (C) HGB, (D) HCT, (E) MCV, (F) MCH, (G) MCHC, (H) RDW, (I) HDW, (J) PLT, (K) MPV, (L) NEU, (M) LYM, (N) MONO, (O) EOS, (P) BASO, (Q) LUC of mice after subacute toxicity study of mumefural. Data are expressed as means ± SD. Figure S2. Serum biochemical parameters (A) AST, (B) ALT, (C) ALP, (D) CPK, (E) TBIL, (F) GLU, (G) TCHO, (H) TG, (I) TP, (J) ALB, (K) A/G ratio, (L) BUN, (M) CRE, (N) IP, (O) Ca^2+^, (P) Na^+^, (Q) K^+^, (R) Cl^−^ of mice after subacute toxicity study of mumefural. Data are expressed as means ± SD. * Significant difference at *p* < 0.05. ** Significant difference at *p* < 0.01 compared with vehicle control group. Figure S3. Absolute organ weights for left adrenal gland, right adrenal gland, thymus, spleen, left kidney, right kidney, heart, lung, and brain of mice after subacute toxicity study of mumefural. Data are expressed as means ± SD. * Significant difference at *p* < 0.05 compared with vehicle control group.
