# Supplementary material for: Acute and Subacute Oral Toxicity of Mumefural, Bioactive Compound Derived from Processed Fruit of Prunus mume Sieb. et Zucc., in ICR Mice
Source: Nutrients. 2020 May 7;12(5):1328. doi: 10.3390/nu12051328 (PMC7284477; doi:10.3390/nu12051328)
Supplement: Supplementary file 1 [file nutrients-12-01328-s001.pdf]

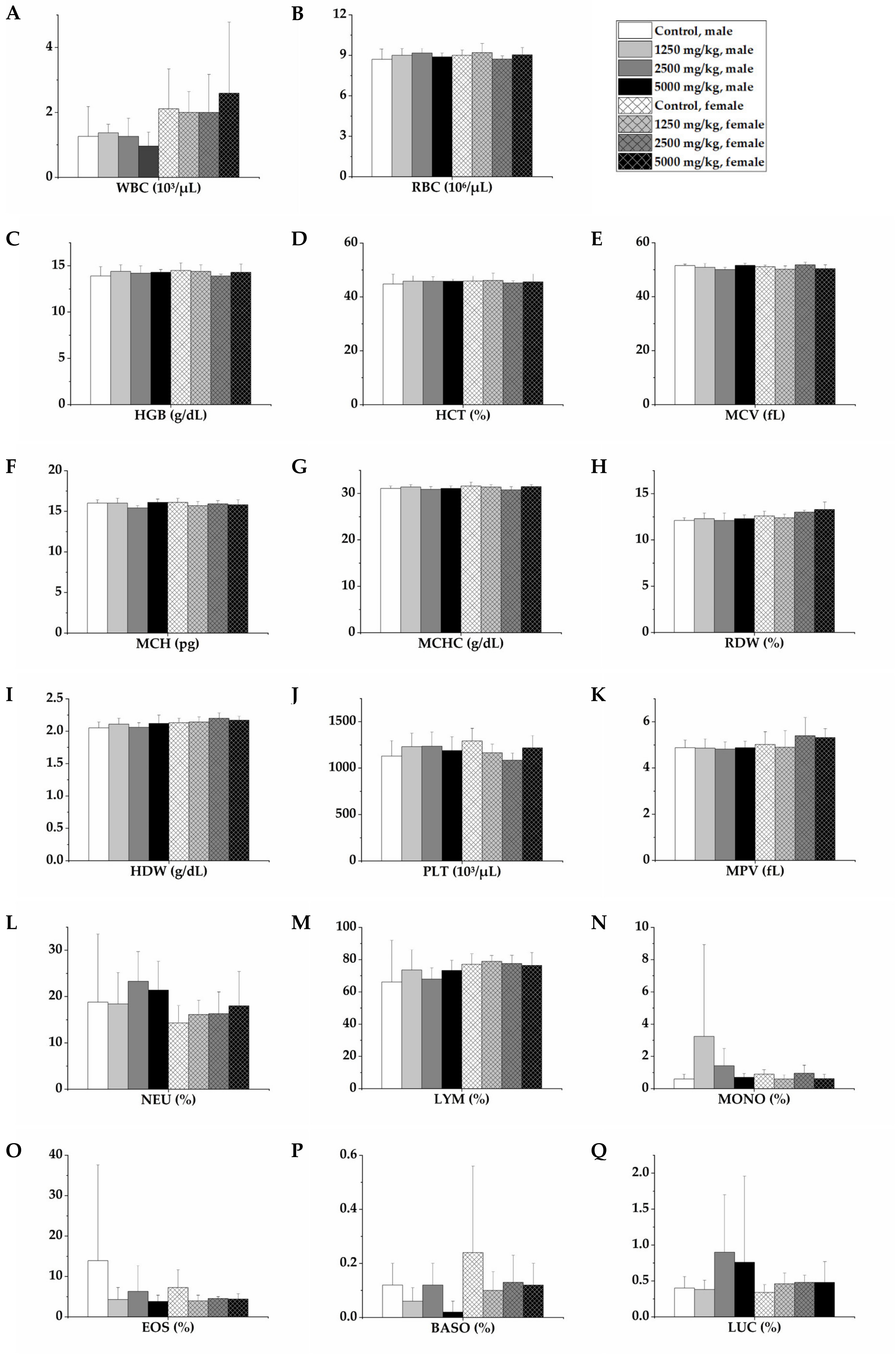

A
WBC ($10^3/\mu L$)
B
RBC ($10^6/\mu L$)
Control, male
1250 mg/kg, male
2500 mg/kg, male
5000 mg/kg, male
Control, female
1250 mg/kg, female
2500 mg/kg, female
5000 mg/kg, female
C
HGB (g/dL)
D
HCT (%)
E
MCV (fL)
F
MCH (pg)
G
MCHC (g/dL)
H
RDW (%)
I
HDW (g/dL)
J
PLT ($10^3/\mu L$)
K
MPV (fL)
L
NEU (%)
M
LYM (%)
N
MONO (%)
O
EOS (%)
P
BASO (%)
Q
LUC (%)

Figure S1. Hematological parameters (A) WBC, (B) RBC, (C) HGB, (D) HCT, (E) MCV, (F) MCH, (G) MCHC, (H) RDW, (I) HDW, (J) PLT, (K) MPV, (L) NEU, (M) LYM, (N) MONO, (O) EOS, (P) BASO, (Q) LUC of mice after subacute toxicity study of mumefural. Data are expressed as means ± SD.

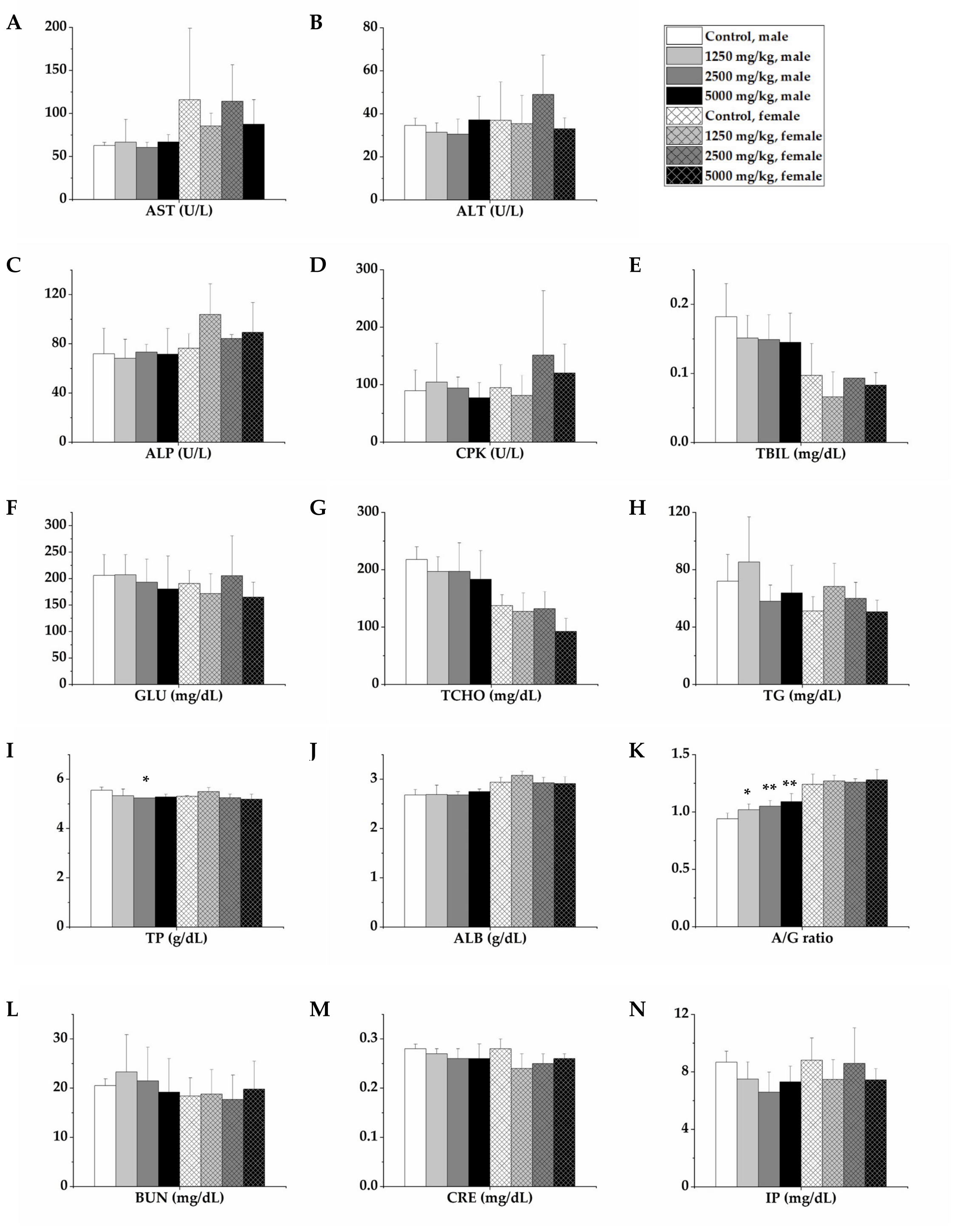

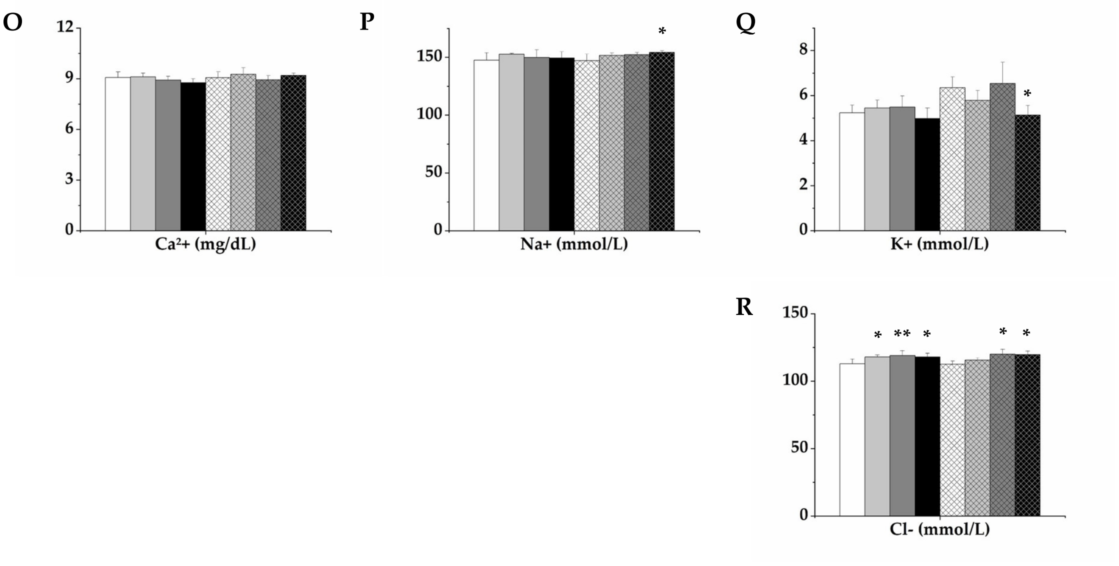

Figure S2. Serum biochemical parameters (A) AST, (B) ALT, (C) ALP, (D) CPK, (E) TBIL, (F) GLU, (G) TCHO, (H) TG, (I) TP, (J) ALB, (K) A/G ratio, (L) BUN, (M) CRE, (N) IP, (O) Ca^{2+}, (P) Na^{+}, (Q) K^{+}, (R) Cl^{-} of mice after subacute toxicity study of mumefural. Data are expressed as means ± SD. *Significant difference at $p < 0.05$. **Significant difference at $p < 0.01$ compared with vehicle control group.

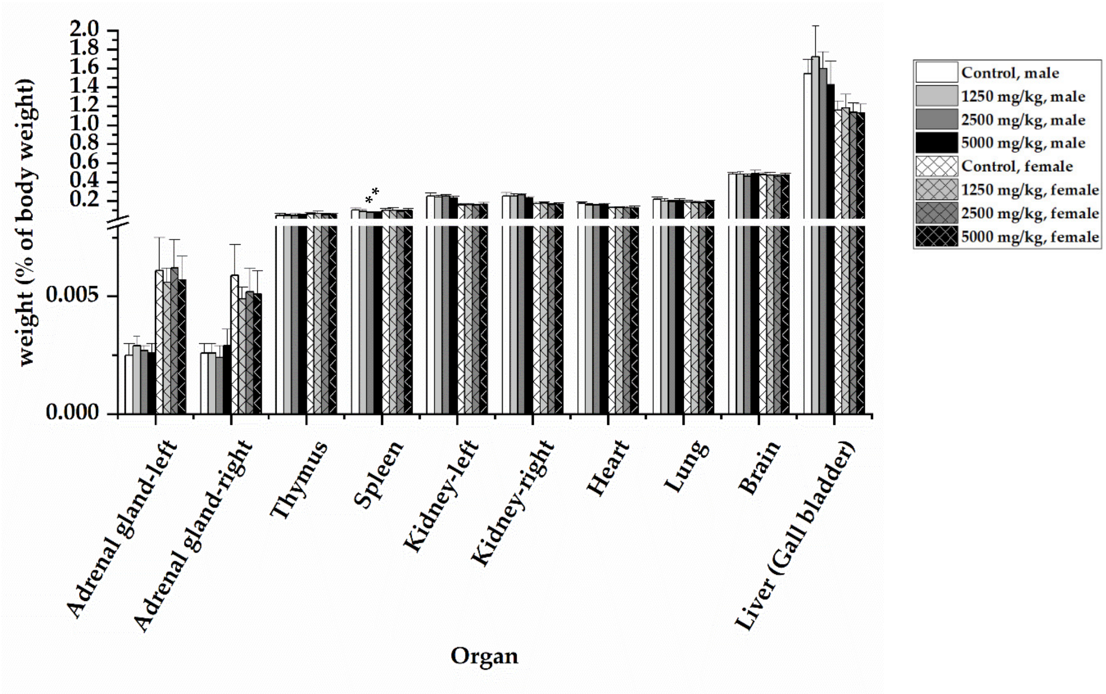

Figure S3. Absolute organ weights for left adrenal gland, right adrenal gland, thymus, spleen, left kidney, right kidney, heart, lung, and brain of mice after subacute toxicity study of mumefural. Data are expressed as means ± SD. *Significant difference at $p < 0.05$ compared with vehicle control group.
